# High level of persister frequency in clinical staphylococcal isolates

**DOI:** 10.1186/s12866-022-02529-7

**Published:** 2022-04-21

**Authors:** Sarita Manandhar, Anjana Singh, Ajit Varma, Shanti Pandey, Neeraj Shrivastava

**Affiliations:** 1 Tri-Chandra Multiple College, Tribhuvan University, Kathmandu, Nepal; 2 Central Department of Microbiology, Tribhuvan University, Kathmandu, Nepal; 3 Amity Institute of Microbial Technology, Amity University, Uttar Pradesh, Noida, UP 201303 India; 4 The University of Southern Mississippi, Hattiesburg, MS-39406 USA

**Keywords:** Biofilm, Persister cells, Clinical staphylococcal isolates, *icaAD* genes

## Abstract

**Background:**

*Staphylococcus aureus* is a notorious human pathogen that causes often lethal systemic conditions that are mostly medical device associated biofilm infections. Similarly, coagulase negative staphylococci are emerging as leading pathogen for nosocomial infections owing to their ability to form biofilm on implanted medical equipment. Chronic in nature, these infections are difficult to treat. Such recalcitrance of these infections is caused mainly due to the presence of persister cells, which exhibit transient yet extreme tolerance to antibiotics. Despite tremendous clinical significance, there is lack of studies on persister cells formation among clinical bacterial isolates. Considering the importance of factors influencing persister formation, in this study, we evaluate the association of antibiotic tolerance with biofilm production, antibiotic stress, growth phase, specimen type, and dependency on staphylococcal species. Biofilm formation was detected among 375 clinical staphylococcal isolates by quantitative tissue culture plate method (TCP) and *icaAD* genes by genotypic method. The antibiotic susceptibility was determined by Kirby Bauer disc diffusion method while minimum inhibitory concentration values were obtained by agar dilution method. Persister cells were measured in the susceptible staphylococcal isolates in the presence of clinically relevant antibiotics.

**Results:**

In the study, 161 (43%) *S. aureus* and 214 (57%) coagulase negative staphylococci (CNS) were isolated from different clinical samples. TCP method detected biofilm production in 84 (52.2%) *S. aureus* and 90 (42.1%) CNS isolates. The genotypic method detected *icaAD* genes in 86 (22.9%) isolates. Majority (> 90%) of both the biofilm producers and non-producers were sensitive to chloramphenicol and tetracycline but were resistant to penicillin. Interestingly, all isolates were sensitive to vancomycin irrespective of biofilm production. While high persister frequency was observed among all staphylococci isolates in the stationary growth phase, the persister frequency in exponential growth phase was statistically high among isolates possessing *icaAD* genes compared to *icaAD* negative isolates.

**Conclusion:**

The research findings provide strong evidence that the clinical staphylococcal isolates exhibit extreme antibiotic tolerance suggesting their causal link with treatment failures. Understanding the factors influencing the formation and maintenance of persister cells are of utmost important aspect to design therapeutics and control recalcitrant bacterial infections.

**Supplementary Information:**

The online version contains supplementary material available at 10.1186/s12866-022-02529-7.

## Background

Persister cells represent a bacterial subpopulation that exhibits transient non-growing state accompanied by transient yet extreme antibiotic tolerance. In 1944, Bigger discovered and coined the term “persister cells” to represent a small fraction of *Staphylococcus aureus* cells that could not be killed by lethal concentration of penicillin [1]. Since last decade, increasing number of studies has highlighted the clinical significance of persister cells in major bacterial pathogens including *S. aureus* and coagulase negative staphylococci (CNS)*.* Persisters during *in vitro* antibiotic treatment display a characteristic biphasic killing curve as majority of the susceptible cells are rapidly killed on exposure to high concentration of antibiotics while small proportion survives for extended time [2, 3]. Upon removal of the antibiotic stress, however, persisters resume their growth and form a population that is identical to the original population in genetic diversity [4] and constituting similar fractions of antibiotic tolerant population [5]. Contrary to the development of antibiotic resistance, antibiotic tolerance in bacteria occurs without any genetic mutation [6]*.* Due to their ability to regrow when treatment is ceased, persister cells can cause the relapse of bacterial infections [7, 8]. This phenomenon, therefore, posits for a paradox that antibiotic susceptible bacteria are the major cause of treatment failures. Besides, persisters are also considered as an important arsenal of bacterial pathogens that allow for their adaptation in stressful environment subsequently increasing the risk of developing antibiotic resistance during treatment [9].

Despite plethora of studies, the mechanisms underlying the persister phenomenon is not yet conclusive. The involvement of stringent response and dormancy were widely recognized as underlying factors for persister formation; however, recent studies have challenged these findings [7, 10–12]. Therefore, the persistence remains an elusive bacterial phenomenon. Nonetheless, a recent study depicts a clinical relevance of treatment failures of *S. aureus* by demonstrating the occurrence of persister switch intracellularly in response to an antibiotic stress [13]. Likewise, high persister phenotype was also observed in clinical *S. aureus* isolates suggesting their importance in treatment management [14]. 

*S. aureus* is a notorious human pathogen that can cause minor skin infections to systemic diseases including infective endocarditis, osteomyelitis and bacteraemia [15]. CNS being the normal commensal of skin and anterior nares have long been considered as non-pathogenic and rarely reported to cause severe infection. However, as a result of the increased use of intravascular devices as treatment as well as increased number of immune compromised patients, CNS has been emerged as a major cause of nosocomial infections [16]. These are mostly biofilm associated infections caused due to the contaminated indwelling devices such as catheters, prostheses, and heart valves that are highly prevalent among health care settings and impose high socio-economic burden worldwide [17]. Bacterial biofilms, which are micro-colonies encased in extracellular polysaccharide material, mediated by gene products of the *icaADBC* operon, are the sources of many bacterial infections which hardly respond to routine treatments. The *icaADBC* encodes four genes including *icaA, icaB, icaC*, and *icaD*, which collectively produce polysaccharide intercellular adhesin (PIA), facilitate the cells binding together and forming into biofilms [18]. Due to physiological stresses resulting through nutrition depletion and oxygen limitation [19], biofilms favour the generation of antibiotic tolerant persister cells, thereby facilitates the recalcitrance of infections [20, 21]. According to the Center for Disease Control and Prevention (CDC), majority of the infections are associated with biofilms formed by *S. aureus, S. epidermidis* and *Pseudomonas aeruginosa* [22, 23]. Therefore, given their contribution in persister generation and subsequent refractory infections, understanding the mechanisms to eradicate biofilms would be a highly remarkable strategy to minimize the risk of treatment failures.

Antibiotic resistance is widespread and imposes a high socio-economic burden. Studies so far have reported high prevalence of antibiotic resistance especially in staphylococcal infections in Nepal [24–28]. In a recent report, we showed the association of biofilm production and antibiotic resistance in the clinical staphylococcal isolates collected in tertiary care hospitals in Nepal [29, 30]. Majority of the isolates (80%) were methicillin resistant *S. aureus* (MRSA) isolates whereas all the isolates were susceptible towards vancomycin. Similarly, almost all the isolates were sensitive towards chloramphenicol and tetracycline (97% and 94%). Likewise, more than 67% isolates were also found to be gentamycin sensitive. Despite high clinical significance of persister cells in treatment failures, there is lack of *in vitro* persister study among *S. aureus* clinical isolates. Given the tremendous importance of antibiotic tolerance and treatment failures, we have investigated the association between persister cells with their plausible determinants: biofilm, antibiotic stress, specimen type (device associated vs others systemic infections) and staphylococcal species (*S. aureus* vs coagulase negative staphylococci, CNS). The antibiotic susceptible staphylococcal isolates that were found to produce *in vitro* biofilms (strong, moderate, weak) by tissue culture plate (TCP) method were randomly selected to measure the persister frequency. Altogether, we tested 60 staphylococcal clinical isolates that comprised ten isolates susceptible to each of the six antibiotics representing different classes of antibiotics: oxacillin, penicillin, vancomycin (ß-lactams), gentamycin (aminoglycoside), ciprofloxacin (fluoroquinolone), and cotrimoxazole. To exclude any possible discrepancy and bias, antibiotic tolerance was measured until 72 h post exposure irrespective of the growth phase or antibiotic used in all the isolates tested. This is the first study in Nepal that investigates the antibiotic tolerance in the clinical staphylococcal isolates.

## Results

### Distribution of staphylococcal species among clinical specimens

In our previous study, we identified 375 staphylococcal isolates in different clinical specimens received in the Microbiology laboratory of two tertiary care hospitals of Nepal. Among these, we identified 161 (43%) isolates as *S. aureus* and 214 (57%) isolates as CNS [30]. Half (*n* = 191, 50.9%) of the isolates were from wound/pus specimens followed by blood (*n* = 90, 24%), urine (*n* = 24, 6.4%), and tips (*n* = 79, 21.1%) (Fig. S1). Likewise, most of the *S. aureus* were isolated from wound/pus (*n* = 135, 36%) specimens whereas lesser number of *S. aureus* isolates were identified in CVC tip (*n* = 9, 2.4%), blood (*n* = 9, 2.1%), catheter tip (*n* = 5, 1.3%) and none from suction tip, endotracheal tube tip (ET) and urine. Unlike *S. aureus*, most of the CNS were identified from blood (*n* = 82, 21.9%) followed by wound/pus (*n* = 56, 14.9%), catheter tip (*n* = 25, 6.7%), urine (*n* = 24, 6.4%), and CVC tip (*n* = 20, 5.3%) (Fig. S1).

### Determination of antibiotic susceptibility profile and minimum inhibitory concentration (MIC) of staphylococcal isolates

Clinically relevant nine different antibiotics belonging to various classes were tested for susceptibility profile via disc diffusion assay. The biofilm productions in these isolates were detected by tissue culture plate method (TCP)—a phenotypic assay, and by detecting *icaAD* genes—a genotypic screening. The results showed that majority of the biofilm producing isolates detected by TCP method were sensitive towards chloramphenicol (94.8%) followed by tetracycline (92%), clindamycin (79.9%), gentamycin (73.6%) and the least number of isolates being susceptible towards penicillin (8.0%). Similar trend of susceptibility was observed in biofilm non-producing isolates (Table 1). Interestingly, the *icaAD* positive isolates also showed similar susceptibility profile such as majority of these isolates were sensitive towards chloramphenicol (94.2%) followed by tetracycline (93%) and clindamycin (86%). Likewise, *icaAD* negative isolates also showed similar trend of the susceptibility towards different antibiotics (Table 1).

**Table 1:** Antibiotic susceptibility profile of biofilm producing staphylococcal isolates by disc diffusion method

**Antibiotics**	**Biofilm producer** **(*n* = 174)**		**Biofilm non producer** **(*n* = 201)**		***icaAD* positive** **(*n* = 86)**		***icaAD* negative** **(*n* = 289)**	
	**Susceptible**	**Resistant**	**Susceptible**	**Resistant**	**Susceptible**	**Resistant**	**Susceptible**	**Resistant**
Cotrimoxazole	94 (54.0%)	80 (46.0%)	111 (55.2%)	90 (44.8%)	48 (55.8%)	38 (44.2%)	132 (45.7%)	157 (54.3%)
Erythromycin	46 (26.4%)	128 (73.6%)	47 (23.4%)	154 (76.6%)	32 (37.2%)	54 (62.8%)	61 (21.1%)	228 (78.9%)
Chloramphenicol	165 (94.8%)	9 (5.2%)	187 (93.0%)	14 (7.0%)	81 (94.2%)	5 (5.8%)	271 (93.8%)	18 (6.2%)
Tetracycline	160 (92.9%)	14 (8.0%)	179 (89.1%)	22 (10.9%)	80 (93.0%)	6 (7.0%)	259 (89.6%)	30 (10.4%)
Clindamycin	139 (79.9%)	35 (20.1%)	157 (78.1%)	44 (21.9%)	74 (86.0%)	12 (14.0%)	222 (76.8%)	67 (23.2%)
Penicillin	14 (8.0%)	160 (92.0%)	12 (6.0%)	189 (94.0%)	6 (7.0%)	80 (93.0%)	20 (6.9%)	269 (93.1%)
Ciprofloxacin	89 (51.1%)	85 (48.9%)	91 (45.3%)	110 (54.7%)	42 (48.8%)	44 (51.2%)	138 (47.8%)	151 (52.2%)
Cefoxitin	46 (26.4%)	128 (73.6%)	55 (27.4%)	146 (72.6%)	29 (33.7%)	57 (66.3%)	72 (24.9%)	217 (75.1%)
Gentamicin	128 (73.6%)	48 (26.4%)	135 (67.2%)	66 (32.8%)	58 (67.4%)	28 (32.6%)	205 (70.9%)	84 (29.1%)

Disc diffusion assay was used to test the antibiotic susceptibility profile of biofilm producing and non-producing isolates.

In our previous study [29], while detecting biofilms, the findings from TCP method best correlated with the *icaAD* genes. The TCP method detected biofilm production in 84 (52.2%) *S. aureus* and 90 (42.1%) CNS isolates, while the PCR detected *icaAD* genes in 86 (22.9%) staphylococcal isolates suggesting their ability to produce biofilms [29]. Furthermore on evaluation of the antibiotic resistance, biofilm producing isolates were found to be resistant towards more number of antibiotics tested indicating association of biofilms with increased antibiotic resistance in the staphylococcal isolates [30]. Herein, we report the MIC values of both biofilm producers and non-producers (Tables 2 and 3).

**Table 2:** Minimum inhibitory concentration (MIC) values of biofilm forming and non-forming isolates as detected by TCP method

Antibiotics	Biofilm			Antibiotics	Biofilm		
	Positive (*n* = 86)	Negative (*n* = 289)	*P* value		Positive (*n* = 86)	Negative (*n* = 289)	*P* value
Oxacillin				Tetracycline			
Range	0.125–64	0.125–64		Range	0.125–8	0.125–8	
MIC_50_	1	1		MIC_50_	0.5	0.5	
MIC_90_	64	64		MIC_90_	2	8	
% resistance	65 (37.4%)	93 (46.3%)	*	% resistance	8 (4.6%)	22 (10.9%)	0.076
Vancomycin				Clindamycin			
Range	0.125–8	0.125–8		Range	0.125–1024	0.125–1024	
MIC_50_	2	2		MIC_50_	0.125	0.125	
MIC_90_	4	4		MIC_90_	256	128	
% resistance	-	-	*	% resistance	32 (18.4%)	36 (17.9%)	0.479
Cotrimoxazole				Penicillin			
Range	0.5/9.5–16/304	0.5/9.5–16/304		Range	0.125–8	0.125–8	
MIC_50_	2/38	16		MIC_50_	0.25	0.5	
MIC_90_	16/304	512		MIC_90_	8	4	
% resistance	78 (44.8%)	76 (37.8%)	0.265	% resistance	132 (75.9%)	163 (81.1%)	0.017
Erythromycin				Ciprofloxacin			
Range	0.125–1024	0.125–1024		Range	0.125–256	0.125–256	
MIC_50_	16	64		MIC_50_	8	8	
MIC_90_	1024	1024		MIC_90_	128	128	
% resistance	102 (58.6%)	139 (69.2%)	0.375	% resistance	104 (59.8%)	115 (57.2%)	0.985
Chloramphenicol				Gentamicin			
Range	0.125–32	1–32		Range	0.125–128	0.125–128	
MIC_50_	4	4		MIC_50_	1	2	
MIC_90_	8	8		MIC_90_	64	64	
% resistance	7 (4.0%)	12 (6.0%)	0.028	% resistance	81 (46.6%)	98 (48.8%)	0.225

The MICs values of the isolates were determined by the agar dilution method. The sensitivity towards each antibiotic was compared between biofilm forming and non-forming isolates. The Chi-square test was used to evaluate the statistical significance of differences in the results. The *p* value of < 0.05 was considered statistically significant.

*The resistant range of MIC values of oxacillin and vancomycin is different for *S. aureus* and CNS. For this reason, *p* value is not mentioned in the table. The sensitivity of oxacillin (*n* = 45, 58.4%) in biofilm producers (*n* = 77) vs sensitivity (*n* = 38, 45.2%) in biofilm non-producers *S. aureus* was not significantly different (*p* = 0.542). Whereas the sensitivity of oxacillin (*n* = 76, 84.4%) in biofilm producers vs. sensitivity (*n* = 96, 77.4%) in biofilm non-producers CNS were significantly different (*p* = 0.086). Irrespective of whether biofilm producers or not, all the isolates were vancomycin susceptible (Supplementary Table 1).

**Table 3:** Minimum inhibitory concentration (MIC) values of biofilm forming and non-forming isolates as detected by possession of *icaAD* genes

Antibiotics	*icaAD*			Antibiotics	*icaAD*		
	Positive (*n* = 86)	Negative (*n* = 289)	*P* value		Positive (*n* = 86)	Negative (*n* = 289)	*P* value
Oxacillin				Tetracycline			
Range	0.125- > 32	0.125- > 32		Range	0.125- > 4	0.125- > 4	
MIC_50_	2	1		MIC_50_	0.5	0.5	
MIC_90_	> 32	32		MIC_90_	1	2	
% resistance	44.6%	37.2%	*	% resistance	-	-	0.217
Vancomycin				Clindamycin			
Range	0.125- > 4	0.125- > 4		Range	0.125- > 512	0.125- > 512	
MIC_50_	1	2		MIC_50_	0.125	0.125	
MIC_90_	2	4		MIC_90_	8	128	
% resistance	-	-	*	% resistance	10.5%	14.5%	0.069
Cotrimoxazole				Penicillin			
Range	0.5/9.5- > 16/304	0.5/9.5- > 16/304		Range	0.125- > 8	0.125- > 8	
MIC_50_	0.5/9.5	16		MIC_50_	0.25	0.5	
MIC_90_	16/304	> 16/304		MIC_90_	2	2	
% resistance	32.6%	42.9%	0.045	% resistance	-	-	0.023
Erythromycin				Ciprofloxacin			
Range	0.125- > 512	0.125- > 512		Range	0.125–520	0.125–520	
MIC_50_	32	32		MIC_50_	8	4	
MIC_90_	512	> 512		MIC_90_	64	128	
% resistance	59.3%	63.3%	0.003	% resistance	46.5%	24.9%	0.015
Chloramphenicol				Gentamicin			
Range	0.125- > 16	1- > 16		Range	0.125- > 64	0.125- > 64	
MIC_50_	4	4		MIC_50_	1	1	
MIC_90_	8	8		MIC_90_	64	64	
% resistance	3.8%	2.3%	0.516	% resistance	23.3%	26.6%	0.231

The MICs values of were determined by the agar dilution method. The sensitivity towards the antibiotics was evaluated among biofilm forming and non-forming isolates based on *icaAD* genes. The Chi-square test was performed to see the association between MIC value and biofilm formation ability taking *P* < 0.05 statistically significant.

*The resistant range of MIC values of oxacillin and vancomycin is different for *S. aureus* and CNS. For this reason, *p* value is not mentioned in the table. The sensitivity of oxacillin (*n* = 20, 44.4%) in biofilm producers (*n* = 45) vs sensitivity (*n* = 63, 54.3%) in biofilm non-producers *S. aureus* was significantly different (*p* = 0.006). Likewise, the sensitivity of oxacillin (*n* = 30, 73.2%) in biofilm producers (*n* = 41) vs sensitivity (*n* = 142, 82.1%) in biofilm non-producers (*n* = 173) CNS were significantly different (*p* = 0.012). Irrespective of whether biofilm producers or not, all the isolates were sensitive towards vancomycin (Supplementary Table 2).

### Dependence between persister frequency, growth phase and antibiotic stress

**Fig. 1:**
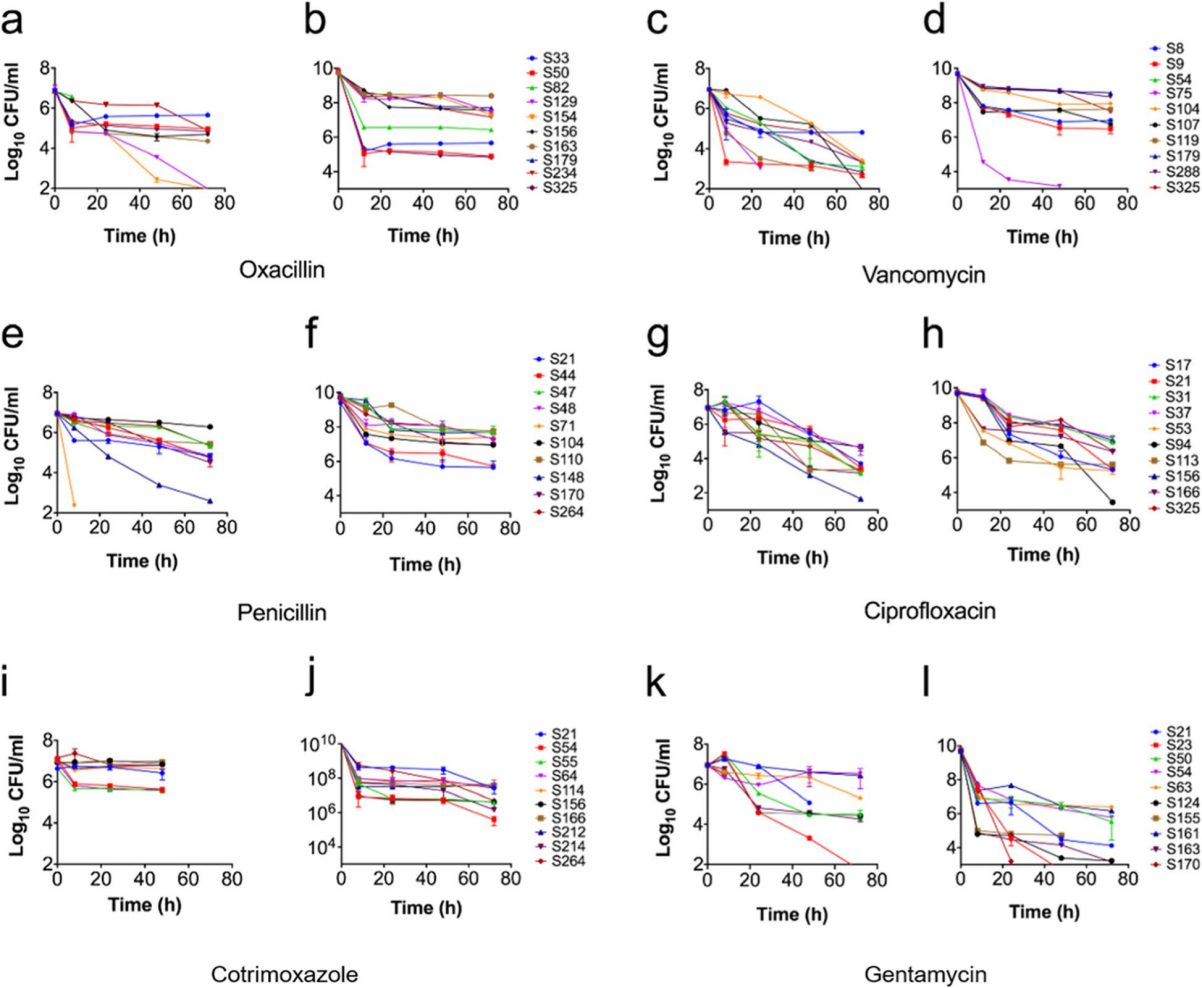
Measurement of persister cells in clinical staphylococcal isolates. Cells grown to exponential phase (**a**, **c**, **e**, **g**, **i** and **k**) and stationary growth phase (**b**, **d**, **f**, **h**, **j** and **l**) were exposed to each antibiotic (20 × MIC for exponential and for stationary growth phase). At designated time points, cells were harvested, washed with PBS and plated for the CFUs count after 24 h of incubation. Data represent the average of three independent experiments. (CNS: S17, S21, S31, S44, S47, S48, S50, S53, S54, S55, S63, S71, S75, S94, S104, S107, S119, S129, S154, S155, S163, S179, S212, S214; *S. aureus*: S8, S9, S23, S33, S37, S64, S82, S110, S113, S114, S124, S148, S156, S161, S166, S170, S234, S264, S288, S325).

The persister frequency among biofilm forming clinical staphylococci isolates were examined in the presence of clinically relevant antibiotics representing different classes. As expected, in the stationary growth phase of bacteria, we observed high persister frequency in almost all the isolates irrespective of antibiotic used (Fig. 1 b, d, f, h, j and l). One clinical isolate (S325) surprisingly failed to form persisters in the presence of vancomycin (Fig. 1d). Considering growth phase dependency of persister fraction, we next sought to examine persister frequency in the exponential growth phase. Although, persister cells were formed during exponential phase, the fraction was lesser as compared to the stationary phase in all antibiotic treatment. Moreover, several staphylococcal isolates against each of the antibiotic stress were defective in persister cells. For instance, three isolates, in the presence of oxacillin (S82, S154, and S234) and vancomycin (S104, S179 and S288) did not form persister cells (Fig. 1a and c). Surprisingly, ciprofloxacin did not eradicate any perister cells in all isolates tested (Fig. 1g and h). Likewise, only a single isolate (S71) failed to form persister in the presence of penicillin (Fig. 1e). Interestingly, all exponentially grown isolates were eradicated within 72 h post-exposure of cotrimoxazole (Fig. 1i). Among those eradicated by cotrimoxazole, one isolate (S156) formed persister in the presence of oxacillin (Fig. 1a and b), while another isolate (S54) failed to form persister in the presence of gentamycin (Fig. 1k).

Altogether, our results showed that the persister phenomenon was observed in both *S. aureus* and CNS. However, it is surprising that, each antibiotic tested could eradicate the persister cells in these staphylococcal isolates. While the deterministic mechanism behind the persister formation and killing remain unclear, the varying effects of persister killing in our study suggest that the type of antibiotic stress largely influence the persister fraction.

### Persister frequency dependent on biofilm production (*icaAD* genes)

**Fig. 2:**
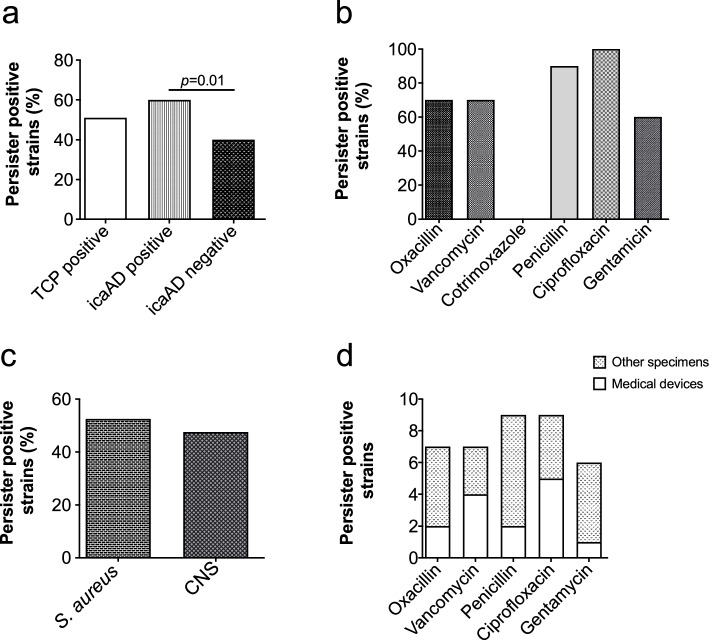
Evaluation of persister frequency on different parameters. **a** Comparison of percentage of persister forming isolates in biofilm producing and non-producing isolates. Persister cells were formed by 51% of TCP positive isolates, while 60% of persister forming isolates was *icaAD* positive, which is statistically different than that of *icaAD* negative isolates. **b** Persister frequency dependency on antibiotic stress used. **c** Persister frequency on device (catheter, CVC, stent) associated isolates and other specimens (blood, urine, wound/pus). **d** Staphylococcal species (*S. aureus* vs CNS). The Chi-square test was used to evaluate the statistical significance of differences in the results. The *p* value of < 0.05 was considered statistically significant

Staphylococcal systemic infections are often caused by device associated biofilms that harbour antibiotic tolerant persister cells. Considering the widespread burden due to the recalcitrance of these infections, we next evaluated the persister fraction based on *in vitro* biofilm production. More than half (51%) of the biofilm forming isolates (as detected by phenotypic TCP assay) formed persister cells in planktonic condition. Further, to evaluate the involvement of *icaAD* genes in persister formation, we compared persister frequency between isolates harbouring *icaAD* genes with *icaAD* negative isolates. Interestingly, we observed higher number of *icaAD* positive isolates formed persister cells as compared to the *icaAD* negative isolates (Fig. 2a), which was statistically significant (*p* < 0.01), suggesting that *icaAD* gene may determine the formation of persister cells. Next, we evaluated whether the nature of biofilm (robust or weak) influences the persister formation in these clinical isolates. Among these, only 13 (46.42%) isolates that produced strong biofilm formed persisters. Similarly, 10 (55.5%) among moderate and 25 (46.2%) among weak biofilm producing isolates formed persisters. These results suggest that persister formation in clinical staphylococcal isolates is likely to occur irrespective of the nature of biofilm (robust vs weak, *p* = 0.78) detected *in vitro* assays.

### Persister frequency dependent on antibiotic stress

Studies on antibiotic persistence mostly use antibiotics that are clinically relevant for pathogen. In this study, to examine whether persister formation depends on antibiotic stress, we used ten antibiotics (belonging to different classes) that have been used to treat staphylococcal infections. Ten clinical isolates that were sensitive to particular antibiotic were tested for persister generation. In these tests, we observed no persister formation in the presence of cotrimoxazole, chloramphenicol and erythromycin. Persister formation was observed in more than half isolates in the presence of vancomycin, oxacillin, ciprofloxacin and penicillin. Gentamycin stress on the other hand produced persister in less number of isolates (Fig. 2b).

### Persister frequency dependent on the type of clinical specimens

The staphylococcal systemic infections characteristically are refractory to the antibiotic treatment. Such recalcitrance is mainly due to the biofilm that mostly harbors antibiotic tolerant bacterial population. Considering this nature of staphylococcal infections in treatment failures, we sought to evaluate whether the persister frequency is influenced by the specimen type of the clinical isolates. We classified all the isolates from devices (catheters, CVC, stent, tips) as medical device associated and those isolated from blood, wound /pus, urine as the other specimen. And evaluation of the results showed no significant difference (*p* < 0.8) in persister formation between device associated staphylococcal isolates with that of other specimens (Fig. 2d). These observations led to an insight that the staphylococcal systemic infections are refractory to the antibiotic treatment irrespective of whether they are device associated biofilm infection or not.

### Persister frequency dependent on the staphylococcal species

Even though CNS also causes biofilm associated infection, almost all persister studies so far have focused on the *S. aureus*. Moreover, in our previous study, we observed high prevalence of antibiotic resistance in CNS [30]. These observations led us to evaluate persister forming ability in CNS as compared to *S. aureus* and determine the persister formation. Interestingly, the results showed similar persister frequency (number of persister positive isolates among total isolates tested) in *S. aureus* and CNS isolates (*p* = 0.54) suggesting the involvement of CNS in treatment failures (Fig. 2c).

## Discussion

In the absence of antibiotic resistance, antibiotic tolerance largely causes the treatment failures of bacterial infections. Due to their importance in public health, large number of study has conducted to understand the mechanism underlying their formation and killing. However, the majority of studies have been conducted using laboratory strains. Lack of studies on clinical isolates has limited our understanding about the persister cells during infections. Therefore, in the present study, we aimed to understand the role of different factors including antibiotic stress, growth phase, species and specimen types in persister formation frequency. Owing to the importance of biofilm in persister tolerance, we also compared the association between biofilm forming ability of clinical staphylococcal isolates and persister formation. *In vitro* experiments show increased persister population as the culture enters the stationary phase [31]. However, small fractions of the persister also form in exponential phase so we examined *in vitro* persister formation in both growth phases of the staphylococcal clinical isolates. To avoid possible interference due to the growth condition of culture, we employed the most widely used persister measurement method in our study. We started the culture with frozen inoculum and standardized the initial culture for both exponential and stationary phase (see method). The cells were grown in 5 ml broth in 50 ml tubes (1:10 of culture-flask ratio) with continuous aeration (shaking at 225 rpm). Moreover, to examine the biphasic killing curves, we counted the CFUs until 72 h post exposure of antibiotics in both exponential and stationary growth phases.

Consistent with the general phenomenon of persister cells, we observed lesser frequency of antibiotic tolerance in exponentially grown cells as compared to the stationary phase cells, supporting the idea that mechanisms of persister formation in clinical isolates varies dependent on the growth phases. In each bacterial population, persister formation inversely corresponds with the cellular energy state [31–33]. Apparently, decreased persister fraction in exponential growth phase indicates higher energy state of growing cells, while extreme tolerance in stationary phase is due to their low energy state since the killing efficiency of bactericidal antibiotics depends on the active target. Extremely tolerant persister cells are formed in the stationary growth phase characterized by a nutrient depletion that exerts stress to the bacterial cells [31]. This environment emulates the chronic bacterial infection especially associated with the biofilms. Given that the depletion of energy generating TCA enzymes and ATP in a growing population determines switch into persister [31, 32], studies so far have shown formation of persister cells in exponentially grown bacterial pathogens as well including *S. aureus, E. coli* and *P. aeruginosa*. Interestingly, in this study, we observed eradication of 40% of staphylococcal isolates in the exponential growth phase. Considering the stochastic depletion of energy in growing population [32], a universal phenomenon, our results indicate a different mechanism of persister formation in exponentially grown clinical staphylococcal isolates. In a recent study, the dependency of energy state was ruled out as the increased persister fraction in *S. aureus* belongs to the clonal complex 30 (CC30) clone as compared to the CC clones was not associated with decreased ATP content. Rather, it showed a reduced membrane potential as a causal link for the increased persister frequency [14]. Due to the limitation of our study, it remains unclear whether membrane potential influences the degree of antibiotic tolerance in these isolates. This study, nonetheless, shows clinical staphylococcal isolates produce high frequency of persister cells in the planktonic condition irrespective of the growth phases.

Biofilm harbour bacterial population that are extremely tolerant to antibiotics. In this study, more than 50% of biofilm producing isolates as detected with phenotypic assays formed persister cells. More interestingly, significantly higher (*p* < 0.01) number of *icaAD* positive isolates were found to form persister cells as compared to *icaAD* negative isolates, suggesting that *icaAD* gene may be a predictable factor for persister generation. These findings altogether suggest that the *in vitro* biofilm detection could be an important preliminary strategy to speculate the possibility of treatment failures in the absence of antibiotic resistance. Since there is lack of studies of persister phenomenon in clinical isolates, further studies are needed to understand the importance of biofilm detection and its relation to the antibiotic failure due to the persistence of isolates towards antibiotics.

Bactericidal antibiotics kill the cells by disrupting their target; a killing mechanism that requires cellular energy. In slow growing stationary state cells, antibiotics cannot act on the target due to reduced energy level [2, 3]. Our observations demonstrate that stationary state cells are indeed extremely tolerant towards the clinically relevant antibiotics. Our observations in all strains, albeit different degree of antibiotic tolerance, nonetheless, demonstrate that stationary state cells indeed are extremely tolerant irrespective of the antibiotics used. In contrary, the antibiotic tolerance of exponentially growing cells appears to depend on the class of antibiotic used. In the present study, all exponentially grown isolates were killed by cotrimoxazole suggesting the possibility that either the persister cells are formed as a response to a stress exerted by an antibiotic or it depends on the killing mechanism of the antibiotics. If either of these factors is true then, the antibiotic tolerance should have been same for all isolates to a specific antibiotic, which is not the case as we observed different fraction although all cells were killed. All these observations indicate the possibility of yet unidentified genetic determinants that are likely to contribute for the persister formation irrespective of growth phase or antibiotics used. Moreover, these findings also demonstrate that persister phenomenon may not be solely dependent on the energy level of bacterial cells specifically in the clinical staphylococcal isolates. Our results infer towards the possibility that the choice of drug is important in curing the staphylococcal infections. Although, there is possibility that the *in vitro* and *in vivo* factors influence the persister frequency, it was apparently observed that the persister formation was dependent on the class of drug used. These results suggest the possibility of specific stress from a drug could also determine the persister formation.

## Conclusions

This study demonstrated the presence of persister cells at high concentrations in stationary cultures. Even though, high persister frequency was observed in isolates with *icaAD* genes, persister was independent of species and type of specimen from which it was isolated. Given that the measurement of persister against antibiotic stress is universally used method and considering the importance of persister in treatment failures, choice of drug would be a good practice to cure recurring infections especially in the absence of antibiotic resistance. All these results indicate the possibility of other unidentified determinants that trigger the persister formation in the clinical staphylococcal isolates.

## Methods

### Bacterial isolates

A total of 375 clinical staphylococcal isolates were collected from two tertiary care hospitals in Nepal. Staphylococci were isolated from various clinical samples and were identified to the species level of both coagulase positive and negative isolates following the standard guidelines as previously described [34]. The staphylococcal species were isolated from different clinical infections including wound/pus, blood, urine and various used tips.

### *In-vitro* assay for biofilm production

We employed the tissue culture plate (TCP), a quantitative assay that is widely used and considered as the standard for biofilm formation detection as previously described [29, 35]. Bacterial cells from fresh culture were inoculated in tryptic soy broth (TSB) with 1% glucose and incubated for 24 h at 37 °C in stationary condition. After incubation, the culture was diluted (1:100) with fresh TSB medium. From this diluted culture, 0.2 ml was inoculated onto individual wells of sterile, polystyrene, flat bottom tissue culture plates (Tarson, India). TSB without bacterial cells served as negative control to check the sterility and non-specific binding of media. Further, the tissue culture plates were incubated for 24 h at 37 °C. After incubation, the content of each well was gently removed by pipetting slowly and tapping the plates. The wells were washed four times with PBS (pH 7.3) to remove the free-floating planktonic bacteria and air dried. The biofilm cells were then fixed with 2% sodium acetate for 5 min and stained with 1% of crystal violet for 15 min, rinsed thoroughly and repeatedly with deionized water. Optical density (OD) of stained adherent bacteria was determined with a micro ELISA auto reader by taking the absorbance at OD_630nm_ [30]. Experiments for each isolate were performed in triplicates and repeated three times. Biofilm production was categorized as negative, weak and high depending on the OD values of adherent cells such as OD value < 0.120 as negative, those with OD > 0.120 and < 0.240 were regarded as weak biofilm-producers. An OD value > 0.240 was indicative of high biofilm-producing bacterial isolates [36].

### Detection of *ica* genes

The genomic DNA from each isolate was extracted using the DNA extraction Kit (Thermo Fisher), following the manufacturer’s instruction. The sequences of intercellular adhesion (*ica*) genes *icaA* and *icaD* (accession number U43366) were taken from the GenBank sequence of the National Center for Biotechnology Information (NCBI) database. Primers specific for *icaA* and *icaD* were designed by the Primer3 program and were purchased from Solis Biodyne, (Denmark). The primer used for the detection of *icaA* was forward 5’-TCTCTTGCAGGAGCAATCAA-3’ and reverse 5’-TCAGGCACTAACATCCAGCA-3’ primer. The two primers include a 188-bp region. For detection of *icaD*, 5’- ATGGTCAAGCCCAGACAGAG-3’ was used as a forward primer and 5’-CGTGTTTTCAACATTTAATGCAA-3’ was used as a reverse primer with the product size of 198 bp [30].

### Antibiotic susceptibility profile

Antimicrobial susceptibility tests of the isolates were carried out by the disk diffusion method using Mueller–Hinton agar plates. The bacterial suspension maintained to 0.5 McFarland standard was used to inoculate Mueller–Hinton agar plates by swabbing them with a sterile cotton swab. Nine different antimicrobial discs (penicillin, tetracycline, clindamycin, chloramphenicol, cefoxitin, erythromycin, cotrimoxazole, gentamycin and ciprofloxacin) were placed and gently pressed down on the surface of the agar plates. Plates were then incubated aerobically for 18–24 h at 37˚C, after that zones of inhibition were measured and recorded. The results were used to determine the isolates as sensitive, intermediate, and resistant according to the Clinical and Laboratory Standards Institute (CLSI) guidelines [37].

### Measurement of Minimum Inhibitory Concentration (MIC)

Agar dilution method outlined in CLSI document M100-S24 (2014) [37] was performed to determine MIC of each isolate. Ten different antibiotics (oxacillin, vancomycin, penicillin, tetracycline, clindamycin, chloramphenicol, erythromycin, cotrimoxazole, gentamycin and ciprofloxacin) were diluted to concentration ranging from 1.25 µg/ml to 5120 µg/ml. Appropriate dilutions of antibiotics were added to molten Mueller Hinton agar and poured into sterile petri dishes. To solidified media, 5 µl of 1:10 diluted 0.5 McFarland standard bacterial suspensions were spot inoculated. After 24 h of incubation at 35 °C, isolates were categorized based on their breakpoints for resistance according to the recommendations by CLSI [37].

### Persister assay

Persister assay was performed for 60 selected staphylococcal clinical isolates representing 10 susceptible isolates for six antibiotics tested. Overnight bacterial cultures were prepared by inoculating frozen cells into 5 ml of TSB in 50 ml culture tubes, incubated at 37 °C in incubator shaker at 225 rpm. The overnight cultures were diluted 1:10 in fresh TSB medium, incubated further for 2 h and normalized to an optical density at OD_600_ of 0.05 in pre-warmed 10 ml TSB in 125 ml conical flask to use as starting culture. The starting bacterial cultures were grown ~ 2 h until they reached an OD_600_ of 0.5–0.6 and 16 h for exponential phase culture and stationary phase cultures respectively. Persister assay was performed in both the exponential and stationary phase staphylococcal cells. When the culture reached at exponential phase, 100 μl of the culture was taken, serially diluted and spread on the surface of TSA plates to determine the initial colony forming units (CFUs) count. The remaining cultures were challenged with 20 × MIC of individual antibiotics and further incubated at 37 °C with continuous shaking (225 rpm). At designated time points after antibiotic exposure, 100 μl of bacterial suspension was taken, washed with PBS and serially diluted. After that, 10 μl cell suspensions were spotted onto TSA plates and incubated for 24 h for persister count. Same procedure was followed for persister assay in stationary phase culture using 100 × MIC of individual antibiotics.

### Statistical analysis

Results for antibiotic susceptibility were expressed in terms of MIC-50 and MIC-90 to represent that of 50% and 90% of the bacterial growth inhibition respectively. The Chi-square test was used to evaluate the statistical significance of differences in the results. The *p* value of < 0.05 was considered statistically significant.

## Supplementary Information

**Additional file 1:** **Figure S1. **Distribution of clinical staphylococcal isolates based on specimen types.

**Additional file 2:** **Supplementary Table 1.**MIC values of biofilm producing isolates detected by TCP method. **Supplementary Table 2. **MIC values of biofilm producing isolates harbouring* icaAD* genes.

## Data Availability

All data generated or analysed during this study are included in this published article and its supplementary information files.

## Abbreviations

CDC: Center for Disease Control and Prevention
MRSA: Methicillin resistant *Staphylococcus aureus*
TCP: Tissue culture plate method
TSB: Tryptic soy broth
CNS: Coagulase negative Staphylococci
OD: Optical density
NCBI: National center for Biotechnology Information
CLSI: Clinical and Laboratory Standard Institute
MIC: Minimum Inhibitory Concentration
CFU: Colony forming unit
CVC: Central venous catheter
ET: Endotracheal tube tip
