# Supplementary material for: High level of persister frequency in clinical staphylococcal isolates
Source: BMC Microbiol. 2022 Apr 21;22:109. doi: 10.1186/s12866-022-02529-7 (PMC10124895; doi:10.1186/s12866-022-02529-7)
Supplement: Supplementary file 1 — Additional file 1: Figure S1. Distribution of clinical staphylococcal isolates based on specimen types. [file 12866_2022_2529_MOESM1_ESM.docx]

**Figure S1.** Distribution of clinical staphylococcal isolates based on specimen types.
