# Supplementary material for: High level of persister frequency in clinical staphylococcal isolates
Source: BMC Microbiol. 2022 Apr 21;22:109. doi: 10.1186/s12866-022-02529-7 (PMC10124895; doi:10.1186/s12866-022-02529-7)
Supplement: Supplementary file 2 — Additional file 2: Supplementary Table 1.MIC values of biofilm producing isolates detected by TCP method. Supplementary Table 2. MIC values of biofilm producing isolates harbouring icaAD genes. [file 12866_2022_2529_MOESM2_ESM.docx]

**Supplementary Table 1: MIC values of biofilm producing isolates detected by TCP method**

| **Antibiotics used** | ***S. aureus*** | | ***P* value** | **CNS** | | ***P* value** |
| --- | --- | --- | --- | --- | --- | --- |
|  | **Biofilm producer (n=77)** | **Biofilm non producer (n=84)** |  | **Biofilm producer (n=90)** | **Biofilm non producer (n=124)** |  |
| **Oxacillin** |  |  |  |  |  |  |
| **Range** | 0.125-64 | 0.125-64 |  | 0.125-64 | 0.125-64 |  |
| **MIC_50_** | 8 | 2 |  | 2 | 1 |  |
| **MIC_90_** | 64 | 64 |  | 64 | 64 |  |
| **% resistance** | 45 (58.4%) | 38 (45.2%) | 0.542 | 76 (84.4%) | 96 (77.4%) | 0.086 |
| **Vancomycin** |  |  |  |  |  |  |
| **Range** | 0.125-8 | 0.125-8 |  | 0.125-8 | 0.125-8 |  |
| **MIC_50_** | 1 | 1 |  | 2 | 2 |  |
| **MIC_90_** | 2 | 4 |  | 4 | 4 |  |
| **% resistance** | - | - | 0.670 | - | - | 0.371 |

**Supplementary Table 2: MIC values of biofilm producing isolates harbouring *icaAD* genes**

| **Antibiotics used** | ***S. aureus*** | | ***P* value** | **CNS** | | ***P* value** |
| --- | --- | --- | --- | --- | --- | --- |
|  | ***icaAD* positive (n=45)** | ***icaAD* negative (n=116)** |  | ***icaAD* positive (n=41)** | ***icaAD* negative (n=173)** |  |
| **Oxacillin** |  |  |  |  |  |  |
| **Range** | 0.125->32 | 0.125->32 |  | 0.125->32 | 0.125->32 |  |
| **MIC_50_** | 2 | 2 |  | 1 | 1 |  |
| **MIC_90_** | >32 | >32 |  | >32 | >32 |  |
| **% resistance** | 20 (44.4%) | 63 (54.3%) | 0.006 | 30 (73.2%) | 142 (82.1%) | 0.012 |
| **Vancomycin** |  |  |  |  |  |  |
| **Range** | 0.125->4 | 0.125->4 |  | 0.125->4 | 0.125->4 |  |
| **MIC_50_** | 1 | 1 |  | 2 | 2 |  |
| **MIC_90_** | 2 | 2 |  | 4 | 4 |  |
| **% resistance** | - | - | 0.723 | - | - | 0.640 |
